# Trait means or variance—What determines plant species' local and regional occurrence in fragmented dry grasslands?

**DOI:** 10.1002/ece3.7287

**Published:** 2021-03-10

**Authors:** Kolja Bergholz, Klarissa Kober, Florian Jeltsch, Kristina Schmidt, Lina Weiss

**Affiliations:** ^1^ University Potsdam, Plant Ecology & Nature Conservation Potsdam Germany; ^2^ Berlin‐Brandenburg Institute of Advanced Biodiversity Research (BBIB) Berlin Germany; ^3^ Leibniz‐Zentrum für Agrarlandschaftsforschung (ZALF) e. V. Müncheberg Germany

**Keywords:** LMA, niche width, plant functional trait, scale‐dependency, species abundance, trait‐environment relationship

## Abstract

One of the few laws in ecology is that communities consist of few common and many rare taxa. Functional traits may help to identify the underlying mechanisms of this community pattern, since they correlate with different niche dimensions. However, comprehensive studies are missing that investigate the effects of species mean traits (niche position) and intraspecific trait variability (ITV, niche width) on species abundance. In this study, we investigated fragmented dry grasslands to reveal trait‐occurrence relationships in plants at local and regional scales. We predicted that (a) at the local scale, species occurrence is highest for species with intermediate traits, (b) at the regional scale, habitat specialists have a lower species occurrence than generalists, and thus, traits associated with stress‐tolerance have a negative effect on species occurrence, and (c) ITV increases species occurrence irrespective of the scale. We measured three plant functional traits (SLA = specific leaf area, LDMC = leaf dry matter content, plant height) at 21 local dry grassland communities (10 m × 10 m) and analyzed the effect of these traits and their variation on species occurrence. At the local scale, mean LDMC had a positive effect on species occurrence, indicating that stress‐tolerant species are the most abundant rather than species with intermediate traits (hypothesis 1). We found limited support for lower specialist occurrence at the regional scale (hypothesis 2). Further, ITV of LDMC and plant height had a positive effect on local occurrence supporting hypothesis 3. In contrast, at the regional scale, plants with a higher ITV of plant height were less frequent. We found no evidence that the consideration of phylogenetic relationships in our analyses influenced our findings. In conclusion, both species mean traits (in particular LDMC) and ITV were differently related to species occurrence with respect to spatial scale. Therefore, our study underlines the strong scale‐dependency of trait‐abundance relationships.

## 1 INTRODUCTION

One of the few laws in ecology is that we find only a few common but many rare taxa in a community (McGill et al., 2007; Preston, 1948). The identification of mechanisms responsible for this pattern, that is, to find and understand the determinants of abundance and rarity of species, may help to improve the understanding and thus prediction of community structure and species distributions (Laughlin et al., 2012; McGill et al., 2007).

Functional traits as measurable characteristics of a species that are connected to its performance mirror the functional adaptations of a species to environmental conditions (e.g., Cornwell & Ackerly, 2009; Wright et al., 2004). Therefore, species niches can be described through their functional traits (Violle & Jiang, 2009). In this concept, the trait mean of a species reflects its niche position along environmental gradients and intraspecific trait variability (ITV) the width of the species' niche (Fajardo & Siefert, 2019; Treurnicht et al., 2020; Violle & Jiang, 2009).

Species mean traits were shown to be suitable predictors for species assembly along environmental gradients (Bergholz et al., 2017; Cornwell & Ackerly, 2009; Grime, 2006; May et al., 2013a). Further, they also predict species abundance at a specific site (Laughlin et al., 2012). Theory postulates that species with intermediate trait values are the most abundant ones, because these species are those that are best adapted to the specific environmental conditions and may have the highest competitive ability (Grime, 2006; Laughlin et al., 2012; Rolhauser et al., 2019). However, solid empirical evidence for an unimodal relationship between species mean traits and abundance remains scarce with studies reporting linear and/or opposing relationships (Cornwell & Ackerly, 2010; Lauterbach et al. 2013; Liu et al., 2017; Read et al., 2017).

One reason for inconsistent trait‐abundance relationships may be that studies were conducted on various spatial scales, indicating that different mechanisms determine species abundance (Lauterbach et al., 2013; Münzbergová, 2004). With increasing spatial extent of the study area, regional processes, like colonization ability, may be more important than local competitive interactions or tolerance to specific conditions (Leibold et al., 2004). This is in particular true for fragmented habitats that consist of isolated habitat patches that are scattered within a landscape. The ability to (re‐) colonize an isolated habitat patch depends on the one hand on the species' dispersal traits (e.g., Belinchón et al., 2020) and on the other hand on the distribution of the species within the whole landscape. Generalist species that occur across a wide range of habitats should have a higher chance to (re‐) colonize isolated habitat patches, because the dispersal distance to the next source population is on average shorter compared with habitat specialists that are confined to the specific habitat type. Hence, at the regional scale habitat specialists are often dispersal‐limited in contrast to habitat generalists (Kolk & Naaf, 2015; Miller et al., 2015). Habitat specialists and generalists vary considerably in their species mean traits, for example, leave trait adaptations to drought in dry grasslands (Hodgson et al., 2011). Therefore, traits that increase habitat specificity should have a negative effect on species occurrence at the regional scale.

The effect of ITV on species abundance gained a lot of attraction recently (Fajardo & Siefert, 2019; Khalil et al., 2019; Shen et al., 2019; Treurnicht et al., 2020). While it was long assumed that ITV is negligible in plant communities, it became evident that ITV represents an ecologically important portion of trait variation in plant communities (Crawford et al., 2019; Siefert et al., 2015). Integrating ITV has been shown to improve the predictive power of species abundance models (Laughlin et al., 2012, but see Read et al., 2017). This occurs because ITV may help to avoid competitive exclusion and predation pressure, and, hence, affect community level dynamics (Banitz, 2019; Bolnick et al., 2011; Crawford et al., 2019; Violle et al., 2012). Further, species with a wider range in trait values should be able to survive in more microsites within habitats (Shen et al., 2019; Violle et al., 2012) and have a better chance to establish in a wider range of environmental conditions. In summary, ITV should have a positive effect on species' abundance on the local and the regional scale.

Following these considerations, this study aims to elucidate the relationships of species mean traits and ITV and species' local and regional occurrence. Our study system is isolated dry grassland patches within an intensively used landscape in NE Germany. In particular, we test whether the means or their variances of three key functional traits (specific leaf area, leaf dry matter content, and plant height) that are associated with stress‐tolerance and competitive ability determine species occurrence of overall 43 species on 21 dry grassland plots. Further, we test whether our results are influenced by the species phylogeny. As outlined by Felsenstein (1985), observations in comparative approaches, such as trait‐based studies, are nonindependent from each other due to the phylogenetic relationships between species. Particular traits and the competitive hierarchy may be phylogenetically conserved (Cahill et al., 2008; Godoy et al., 2014), which may influence the observed trait‐occurrence relationships.

Specifically, we hypothesize that:

1. At the local scale, species with intermediate mean traits are the most frequent, because these species are best adapted to the local environmental conditions.
2. At the regional scale, species mean traits that are connected to stress‐tolerance have a negative effect on species occurrence, because dispersal limitation is higher for habitat specialists compared with generalists.
3. At both spatial scales, ITV has a positive effect on species occurrence, because a higher variability in traits increases the niche width and allows to establish under different conditions.

## 2 MATERIAL & METHODS

### 2.1 Study region

**FIGURE 1:**
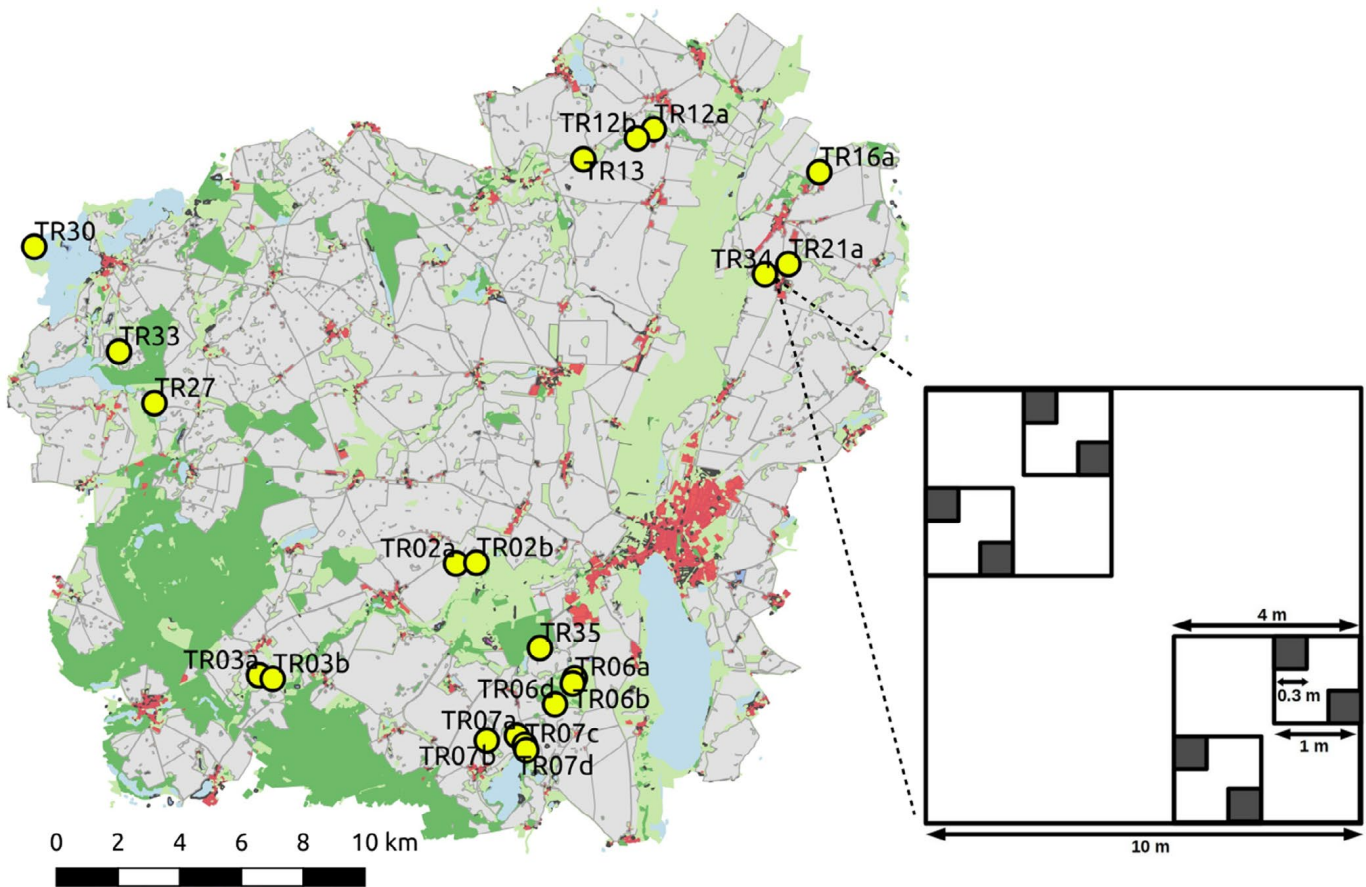
Overview of the study area and sampling design. Most of the landscape is used for agriculture (gray). Forests (dark green) make up to 13% coverage and different types of mesic to intensive grasslands (bright green) about 12%. Dry grasslands cover <1% and appear mainly in small patches. At 21 dry grassland patches (coordinates see Appendix S1), we made nested vegetation surveys within 10 m × 10 m plots including eight 0.3 m × 0.3 m subplots. Regional species occurrence refers to the species occurrence within the 10 m × 10 m plots. Local occurrence refers to the occurrence of the species across the eight subplots within each plot. Trait measurements were conducted for six randomly selected species with 12 individuals in each plot

This study was conducted in the northeastern part of the federal state of Brandenburg in Germany (Figure 1, AgroScapeLab, http://www.zalf.de/de/struktur/eip/Seiten/AgroScapeLab.aspx, 52°52′N—53°23′N, 13°20′E—14°12′E; Wulf et al., 2016). The study area is located at the transition zone of the west‐European oceanic and the east‐European continental climate and is characterized by a temperate climate (8.6°C) with low annual precipitation (563 mm). The region is sparsely populated and to a great extent intensively used for agriculture (62% of the area). Forests make up to 13% of the area and grasslands around 12%. The dominant grassland types in this region are wet, mesic, and intensive grasslands as well as fallows (all around 2%–3%). Dry grasslands are found mainly on hills and slopes or former military areas and make less than one percentage of the land cover. Dry grassland patch sizes vary between 270 and 100.000 m^2^, with a median of 5,600 m^2^. The dry grassland plant communities belong to the class *Festuco‐Brometea* with some elements of the class *Koelerio‐Corynepheretea*. These plant communities developed under the constant land use of humans as pastures for sheep and goats for several hundred years. Nowadays, the selected sites are still grazed or to a lesser extent mown. The species composition is a mixture of *true* dry grassland specialists that are restricted to these habitats (e.g., *Phleum phleoides*, *Dianthus cartusianorum*, *Thymus pulegioides*) and generalists that occur also in disturbed or more mesophile habitats (e.g., *Plantago lanceolata*, *Arrhenatherum elatiu*s, *Carex hirta*). Exotic or invasive species are almost absent on the investigated sites. The natural vegetation on current dry grassland sites would be mainly sparse oak‐dominated forests (Hofmann & Pommer, 2005).

### 2.2 Data collection

Data collection took place from May to July 2017 at 21 dry grassland patches (Figure 1, Appendix S1, Table S1). Within each patch, one 10 m × 10 m plot in a visually homogeneous area was established. At each plot, nested vegetation surveys were conducted including eight 0.3 m × 0.3 m subplots (Figure 1). Local occurrence defines the species frequency within these eight 0.3 m × 0.3 m subplots for each plot. Local occurrence ranges, therefore, from zero to eight. This measure was highly correlated to the estimated species cover values as alternative, but more subjective, species abundance (*r*_spearman_ = 0.83, *p* < .001). Regional species occurrence was defined as the number of 10 m × 10 m plots that a species was found (ranging between 1 and 21).

Trait measurements were conducted within all 21 plots, in order to estimate species mean traits and intraspecific trait variation. At each plot, we randomly chose six perennial species and measured traits of 12 randomly selected mature individuals per species within the plots (i.e., seedlings were excluded from the selection). Hence, we measured traits of 1,521 individuals in total (21 plots × 6 species × 12 individuals). Due to the random selection of species, some species were sampled at different plots. For all individuals, we measured three key functional traits that are correlated to different lifecycle strategies, specific leaf area (SLA), leaf dry matter content (LDMC), and plant height. SLA and LDMC are part of the leaf economic spectrum and capture well the trade‐off between fast‐growing and conservative growth (Diaz et al., 2016; Wright et al., 2004). Plant height is positively correlated with the ability to gain resources, produce biomass, and fitness (Kraft et al., 2015; Moles, 2018; Westoby et al., 2002). Low values of SLA and plant height and high LDMC are associated with dry and nutrient poor conditions (May et al., 2013a; Perez‐Harguindeguy et al., 2013), indicating stress‐tolerance toward the environmental conditions of dry grasslands. We consider therefore these traits typical for dry grassland specialists that should be negatively correlated with regional species occurrence (hypothesis 2). Traits were measured for all selected individuals following Perez‐Harguindeguy et al. (2013). Plant height (cm) was measured as the distance between the highest photosynthetic tissues of a plant and the soil surface. For leaf measurements, two healthy, sun‐exposed leaves per plant were sampled to calculate one average value for each individual. To achieve full turgescence of the leaves, they were stored in tubes with moist paper until leaves were scanned to determine the leaf area and to measure leaf fresh mass (mg). Afterward, leaves were dried in an oven at 80°C for 24 hr to weigh leaf dry mass for LDMC (mg/g) and SLA (mm^2^/mg).

### 2.3 Statistical analyses

We built global models for the local and regional occurrence and assessed whether phylogenetic correction influenced our results with separate models. Local occurrence was modeled with GLMMs with a binomial error‐distribution (*glmer*, R‐package *lme4*, Bates et al., 2015). As response variable, we used the odds of the species occurrence within the eight 0.3 m × 0.3 m subplots within the plots. The odds determine the number of successes and failures (presence and absence of a species in subplot) and are particular useful to model count data that are strictly bounded to a minimum and maximum (see e.g., Crawley, 2007). As predictors, we included the mean and the coefficient of variation of the three traits SLA, LDMC, and plant height, calculated from the 12 measured individuals of the respective plots. The mean trait values were included as linear and quadratic terms and the coefficients of variation as linear term. The quadratic term was included to test the prediction that species with intermediate trait values have the highest species occurrence (hypothesis 1). Plot and species were included as crossed random effects to account for the non‐independence of the data structure. We set up a second model that corrected for the phylogenetic relationship between the species and used a novel approach by Kain and Bolker (2019) for GLMMs. The structure, that is, fixed and random effects, of this phylogeny model was the same as in the former model, but it incorporates phylogenetic correlations by modeling them as random effects (see Kain & Bolker, 2019 for details). We used the *Daphne* tree (Durka & Michalski, 2012) to estimate the phylogenetic distances between species. Multichotomies were resolved in the order they appear in the tree for further analyses.

We used linear models to analyze the regional occurrence of species, that is, the number of species occurrences within the 10 m × 10 m plots at the landscape. Since residuals of the models were normally distributed, we used linear models, though our response variable is strictly speaking count data. In a first step, we needed to estimate trait measures (means and coefficients of variation) for each species for the whole landscape, since we measured traits from different species repeatedly at different sites. For this purpose, we built linear mixed effects models (*lmer*, R‐package lme4) that incorporated species as fixed effect and the local sites as random intercept effect. The predictions of these models served as “landscape‐wide” trait measures and were used as predictors in our linear model. We set up a second regional occurrence model that used the traditional phylogenetic independent contrasts (Felsenstein, 1985) implemented in the R‐package *ape* to reveal how phylogeny influenced our results.

In order to assess how local and regional species occurrence was related to trait measures, we applied an information theoretic approach (Burnham & Anderson, 2002). We constructed a series of models that contained all possible combinations of predictors from the global model (Burnham & Anderson, 2002). The models were compared with each other with the AIC_c_, in order to find the most likely parameter combination that predicts species occurrence. Hereby, it turned out that several models performed equally well, indicating that different models that present alternative hypotheses explain species occurrence equally well. Therefore, we performed model‐averaging of the best models (delta AIC_c_ < 2) to get more reliable parameter estimates (Burnham & Anderson, 2002). We tested whether the cut‐off criterion (delta < 2) affected our main results, which was not the case. Additionally, we calculated the conditional and marginal $R_{\text{GLMM}}^{2}$ (Nakagawa et al., 2017) for best local and adjusted *R* ^2^ for the best regional models. We report averaged *R* ^2^ values across the best models in the main manuscript. All traits were log‐transformed and z‐scaled prior to analyses, in order to meet statistical model assumptions and to allow comparisons among parameter estimates (Schielzeth, 2010). Trait measures were not strongly correlated (cor < 0.5). We excluded *Sedum acre* from our analyses, as the only representative of the *Crassulaceae* family with succulent leaves had outlying LDMC values. The exclusion had no effect on our main findings (Appendix S2). All statistical analyses were conducted in R 3.6.2 (R Core Team, 2019) with the R‐packages *MuMin*, *lme4*, *picante*, *Dharma*, and *ape*.

## 3 RESULTS

We sampled in total 43 species within the 21 10 m × 10 m plots (Appendix S3). The measured species differed in their local occurrence (ranging from 0 to 8, mean ± *SD* = 2.73 ± 2.43) and on the regional scale (ranging from 1 to 20, mean ± *SD* = 8.98 ± 4.83). However, local and regional species occurrence were not correlated to each other (*r* = −.23, *p* = .13). This indicates that locally rare species were not necessarily rare at the regional scale and *vice versa*.

**FIGURE 2:**
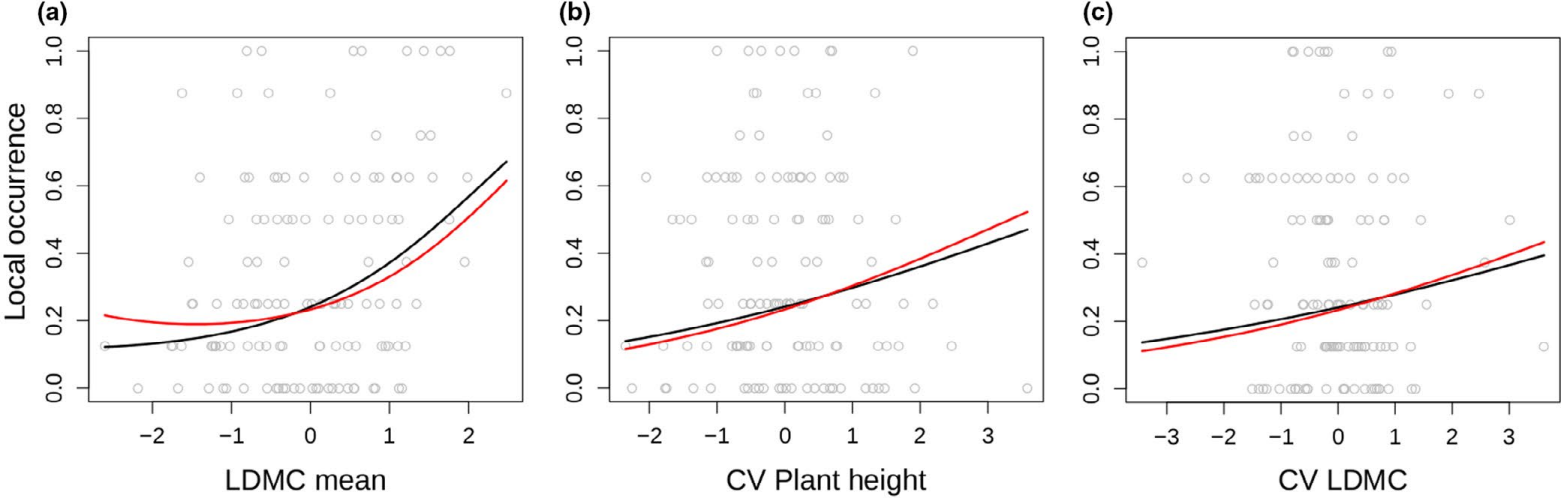
Relationship between traits and local species occurrence: (a) mean leaf dry matter content (LDMC mean), intraspecific variation, measured as the coefficient of variation, of (b) LDMC, and (c) plant height. Lines represent model predictions of the averaged models for the standard GLMM (black line), and the one that incorporates the phylogenetic relationships between species (red line). Grey points refer to the data points. Please note that data points belong to different sampling sites and species

We predicted (hypothesis 1) that species with intermediate traits are the most frequent at the local scale. This hypothesis was not supported by our data, since negative quadratic effects of species mean traits on local species occurrence did appear only ones for SLA within the best models (Appendix S4). The parameter estimate of SLA within the averaged model is very small and can be, therefore, considered as negligible (Table 1). In contrast, the averaged model predicted a positive effect of species mean LDMC on local species occurrence (Figure 2a), indicating that stress‐tolerant species dominate the local community. These results were independent with respect to the phylogenetic correction (Figure 2, Table 1).

**TABLE 1:** Relationship between species occurrence and traits at the local and regional scale.

Phylogenetic correction	Local abundance		Regional abundance	
	No	Yes	No	Yes
Intercept	−1.1 ± 0.27	−1.19 ± 1.16	0.02 ± 0.16	**‐**
Plant height				
Mean			0.02 ± 0.09	
Mean^2^				
CV	**0.29 ± 0.11**	**0.36 ± 0.13**	**−0.45 ± 0.19**	**−0.44 ± 0.15**
LDMC				
Mean	**0.54 ± 0.20**	**0.36 ± 0.21**	−0.04 ± 0.11	**−0.22 ± 0.18**
Mean^2^	**0.09 ± 0.11**	**0.12 ± 0.13**	0.03 ± 0.09	−0.01 ± 0.04
CV	**0.20 ± 0.11**	**0.26 ± 0.11**	0.04 ± 0.13	−0.02 ± 0.08
SLA				
Mean	0.10 ± 0.15			−0.03 ± 0.12
Mean^2^	−0.03 ± 0.06			
CV			−0.02 ± 0.09	−0.01 ± 0.05
*R* ^2^			.10	.17
Marginal	0.11	0.23		
Conditional	0.84	0.62		

Note

The table shows standardized parameter estimates (± standard errors) of the averaged models for both local and regional species occurrences, each for models with or without phylogenetic correction. Bold‐typed estimates indicate that the parameters were included in the best model (lowest AIC_c_). If no parameter estimate is given, the parameter did not appear in any of the best models (see Appendix S4, Table S3 for an overview of the best models). For the regional model with phylogenetic independent contrasts, no intercept is estimated (see Felsenstein, 1985). The reported *R* ^2^ represent averaged values of the best models.

**FIGURE 3:**
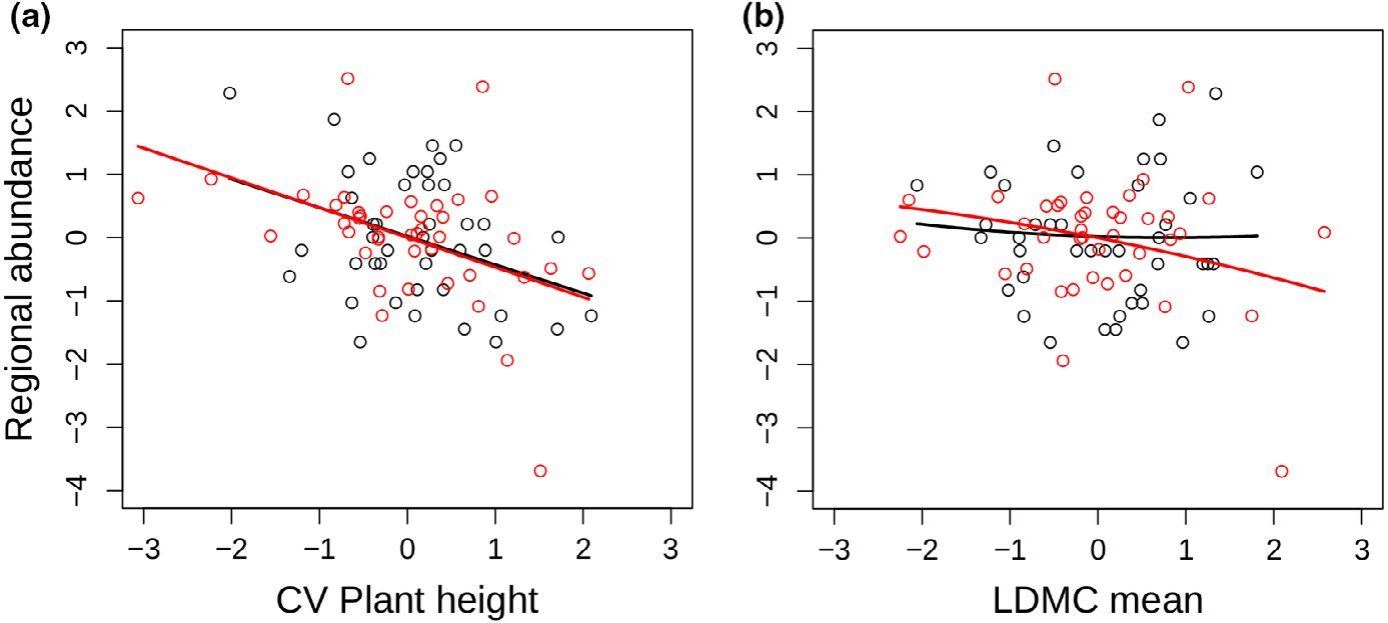
Effect of intraspecific variation of plant height (a) and mean leaf dry matter content (b) on regional species occurrence (both scaled). Black points refer to original data points and the black line represents the prediction of the respective model. Red points represent phylogenetically corrected data points using phylogenetic independent contrasts. The red line represents the respective model prediction

Species mean traits related to stress‐tolerance were predicted to have a negative effect on regional species occurrence (hypothesis 2). Indeed, species mean LDMC was negatively associated with species occurrence in the phylogenetic‐corrected model (Table 1, Figure 3b). However, this relationship was rather weak and was absent in one of the best six models (Appendix S4). Hence, this finding should be taken with caution and gives only limited support for hypothesis 2.

We found mixed evidence that ITV increases species occurrence (hypothesis 3). At the local scale, ITV of LDMC and plant height (Figure 2b,c) had a positive effect on species occurrence supporting hypothesis 3. At the regional scale, species occurrence was, however, negatively related to intraspecific variation of plant height (Figure 3a), indicating that more variable species are less common. We would like to emphasize that this finding was not influenced by our sampling design, that is, that some species were sampled more often than other species due to random selection, as ITV plant height was not correlated with sampling effort (linear model, *F*_1,41_ = 0.95, *p* = .33).

Overall, the explanatory power of our models was higher in the phylogenetic‐corrected models compared with the standard ones (see *R* ^2^ values, Table 1).

## 4 DISCUSSION

In this study, we tested how species mean traits and intraspecific trait variability (ITV) are related to species occurrence at local and regional scales in isolated dry grassland patches.

We found no support that species with intermediate species mean traits are the most common ones in local communities (hypothesis 1). In contrast, we detected a positive effect of mean LDMC on local species occurrence. Plants with high LDMC have a greater tolerance toward physical stress, like water limitation, or disturbance by trampling of grazers as a result of their conservative growth strategy (Hodgson et al., 2011; Perez‐Harguindeguy et al., 2013). Typical dry grassland species, that is, species that are restricted to this habitat, show higher LDMC on average compared with mesophile species (Gross et al., 2007; Hodgson et al., 2011). Hence, our result indicates that habitat specialists rather than species with intermediate species mean traits nor ubiquitous, generalist species dominate the dry grassland communities locally. Moreover, we found no indication that traits related to high competitive ability affect species occurrence at the local scale. Large plant height and low SLA are consistently correlated with competitive ability (Carmona et al., 2019; Kraft et al., 2015; Kunstler et al., 2016), but had no apparent effect on species occurrence in our models. Further, the positive mean LDMC‐species occurrence relationship indicates that even species with a low competitive ability dominate the community, as LDMC is negatively related to competitive ability (Kraft et al., 2015; Rolhauser et al., 2019). One possible explanation for the missing positive effect of traits associated with competitive ability and species occurrence may be reasoned by our study system. Dry grasslands are characterized by a low productivity coupled with disturbance. In such environments competition, particularly above‐ground, should be lower than in more productive habitats (Grime, 2007).

At the regional scale, LDMC was negatively associated with regional species' occurrence, if the phylogenetic relationship between species was considered. Although this finding may be a hint that habitat specialists (i.e., low LDMC) are less common on the regional scale, because of a lower (re‐)colonization ability (hypothesis 2), the relationship was rather weak and other species mean trait‐ occurrence relationships were even less pronounced. Therefore, we conclude that the regional occurrence is primarily driven by other factors than species mean traits related to habitat specificity (see discussion on predictive power below).

Species with a wider niche (large ITV) are more frequent at the local scale, which supports hypothesis 3. We attribute the positive effect of ITV in LDMC and plant height to a higher adaptability of species to local heterogeneity. Dry grasslands show a significant variability in soil conditions, like water, and nutrient availability (Harze et al., 2016), due to variation in soil depth and topography or the excretion by sheep. In addition, sheep create heterogeneity in light availability as the result of (selective) grazing and trampling. Such local heterogeneity provide microhabitats that may select for specific traits and drive the assembly of species (Bergholz et al., 2017). Similarly, we assume that species that are able to adjust their functional traits to different environmental conditions of microhabitats dominate at the local scale. Further, ITV has the potential to lower competitive exclusion (Banitz, 2019; Crawford et al., 2019), as it may reduce competitive hierarchies among species (Carmona et al., 2019). However, this scenario seems unlikely in the absence of any indication that species mean traits linked to competitive ability positively affect species occurrence.

In contrast, we found no evidence that species with a higher ITV are more frequent at the regional scale. Intraspecific variation in plant height had even a negative effect on species occurrence. This finding is very surprising, and we cannot offer a clear interpretation of this result. Intraspecific variation in plant height may indicate that the fitness of individuals differs between sites, as plant height is positively correlated to fitness (Breitschwerdt et al., 2019), and thus, intraspecific variability of plant height may be a measure of how susceptible individuals respond to different environmental conditions. Species that have a large plant height variation may be unsuccessful under certain environmental conditions and therefore rare. This hypothesis is speculative and needs definitely refined empirical evidence.

The low predictive power of our models may indicate that we possibly missed important aspects of the niches, respectively, functional traits. For this study, we used two key functional leaf traits and plant height, because these easy‐measurable traits are fundamentally important to characterize the species niche (Diaz et al., 2016; Wright et al., 2004) and are good predictors for community assembly in similar study systems (e.g., de Bello et al., 2013; Lhotsky et al., 2016). Further, these traits are highly variable within and across species (Siefert et al., 2015), which is particularly appropriate to address our study aim, whether species mean or ITV affects species occurrence. However, since traits associated with above‐ground competitive ability seem to have no effect on local species occurrence and water limitation is a central characteristic of our study system; it seems plausible that below‐ground traits may play a large role for species occurrence in dry grasslands. Root traits, like rooting depth or specific root length, are important measures for water acquisition and were shown to represent different niche axes than above‐ground traits (Bergmann et al., 2017). Hence, these traits may mediate competition for water and nutrients and, therefore, affect local species occurrence. Further, it should be acknowledged that other processes than environmental tolerance or competition among plants structure local plant occurrence, like negative plant‐soil feedbacks, pollination, or herbivory. Relating these mechanisms to plant functional traits and their association to species occurrence may be a reasonable approach. At the regional scale, we are not confident that the inclusion of further traits would improve our model predictions substantially. In preliminary analyses, the consideration of seed mass as additional proxy for dispersal ability beside plant height or the interactions between traits did not explain our regional (and local) data better. Either (re‐) colonization on dry grassland patches is primarily driven by stochastic events rather than by biological traits of species, as predicted by the theory of island biogeography (MacArthur & Wilson, 1967). Or, (re‐) colonization events are less important for species occurrence as expected. May et al. (2013b), for instance, showed that even annual plant communities of less fragmented habitats were better described by an “isolated island” model compared with meta‐community or mainland‐island models. This scenario may also hold for our study system, since seedling establishment in dry grasslands is often hampered by seed or microsite availability (Münzbergová, 2004; Zeiter et al., 2006). Under this consideration, the current regional species occurrence is the outcome of long‐lasting historical processes (see e.g., Münzbergová, 2004), while current regional processes and related traits to these may be negligible.

Our results are unbiased with respect of phylogeny. Whether this pattern is true for other study systems, remains open. Our study system is relatively young in comparison with the species phylogeny. The regional species pool established presumably after the last ice age (~11.000 years), whereas the local species composition of dry grasslands were heavily influenced by man since several hundred years (Poschlod & WallisDeVries, 2002). Study systems in which species evolved jointly over a longer period may show stronger influence with respect to phylogenetic relationships.

## 5 CONCLUSIONS

In conclusion, we found no support that species with an intermediate trait value are those that dominate the grassland plant communities. However, we showed that ITV had a positive effect on species occurrence at the local scale. Since ITV may be a substantial part of trait variation at local scales (Siefert et al., 2015), it may be even more important than a specific mean value. This may be in particular true if local heterogeneity is high (Shen et al., 2019). Our contrasting findings of intraspecific and species mean trait‐occurrence studies underline that the relationships are scale‐dependent, though the findings differed from our predictions. The effect of both ITV and species mean traits on competitive ability or response to environmental heterogeneity may change between study systems. Therefore, we see a strong need to couple trait‐occurrence studies with experiments that quantify trait responses to competition and environmental conditions, in order to reveal the underlying mechanisms that drive species abundance.

## CONFLICT OF INTEREST

The authors declare no conflict of interest.

## AUTHOR CONTRIBUTIONS

**Kolja Bergholz:** Conceptualization (lead); Formal analysis (lead); Methodology (lead); Project administration (supporting); Supervision (lead); Validation (lead); Visualization (lead); Writing‐original draft (lead); Writing‐review & editing (equal). **Klarissa Kober:** Conceptualization (supporting); Data curation (lead); Formal analysis (supporting); Investigation (lead); Methodology (supporting); Validation (supporting); Writing‐original draft (equal); Writing‐review & editing (equal). **Florian Jeltsch:** Funding acquisition (lead); Project administration (supporting); Resources (lead); Supervision (supporting); Writing‐original draft (supporting). **Kristina Schmidt:** Data curation (supporting); Investigation (supporting). **Lina Weiss:** Conceptualization (supporting); Data curation (supporting); Project administration (lead); Supervision (supporting); Writing‐original draft (equal); Writing‐review & editing (supporting).

## Supporting information

Supplementary Material

## Data Availability

We stored all collected data (trait measurements and species occurrence) at the open research database of ZALF (https://doi.org/10.4228/ZALF.DK.146).
