# Supplementary material for: Trait means or variance—What determines plant species' local and regional occurrence in fragmented dry grasslands?
Source: Ecol Evol. 2021 Mar 10;11(7):3357–65. doi: 10.1002/ece3.7287 (PMC8019038; doi:10.1002/ece3.7287)
Supplement: Supplementary file 1 — Supplementary Material [file ECE3-11-3357-s001.docx]

Supplementary Material

**Appendix S1**

**Table S1:** Location of the dry grassland plots in the study area. GPS-Coordinates of the 21 dry grasslands plots used in this study, situated in the north-eastern part of the federal state of Brandenburg in Germany. Coordinates are given in the format decimal degrees (DD).

Plot X-coordinate Y-coordinate

TR02a 13.7561500 53.3071250

TR02b 13.7660500 53.3074610

TR03a 13.6613765 53.2737500

TR03b 13.6680485 53.2728085

TR06a 13.8147655 53.2745205

TR06b 13.8138570 53.2730040

TR06d 13.8054000 53.2669000

TR07a 13.7871375 53.2576025

TR07b 13.7725415 53.2560750

TR07c 13.7909765 53.2550100

TR07d 13.7918485 53.2534765

TR12a 13.8489280 53.4338070

TR12b 13.8407470 53.4310905

TR13 13.8148305 53.4247580

TR16a 13.9299655 53.4221400

TR21a 13.9155585 53.3954165

TR27 13.6079750 53.3519330

TR30 13.5479350 53.3965300

TR33 13.5902550 53.3666650

TR34 13.9042165 53.3923415

TR35 13.7976250 53.2831050

**Appendix S2**

**Table S2:** Relationship between species occurrence and traits at the local and regional scale including *Sedum* *acre*. The table shows standardized parameter estimates of the averaged models for both local and regional species abundance, each for models with or without phylogenetic correction. Bold-typed estimates indicate that the parameters were included in the best model (lowest AICc). For the regional model with phylogenetic independent contrasts, no intercept is estimated (see Felsenstein 1985).

In general, *Sedum acre* did not affect our main findings (see Results, Tab. 1). Some minor differences are detected for the influence of LDMC on species occurrence. Noticeable deviations are marked in red.

Local abundance Regional abundance

Phylo. Corr. No Yes No Yes

Intercept -1.2±0.28 -1.27 ±1.13 0.04 ±0.15 --

**Height**

mean -- -- 0.03 ±0.1 --

mean² -- -- -- --

CV **0.29 ±0.12 0.36 ±0.13 -0.43 ±0.19 -0.44 ±0.15**

**LDMC**

mean **0.66 ±0.24** **0.44 ±0.25** -0.02 ±0.07 **-0.22 ±0.18**

mean² **0.15 ±0.1** **0.14 ±0.12** -- -0.01 ±0.04

CV **0.23 ±0.11** **0.27 ±0.11** 0.05 ±0.13 0.06 ±0.12

**SLA**

mean 0.09 ±0.16 -- -- -0.03±0.11

mean² -0.02 ±0.06 -- -- --

CV -- -- -- -0.01 ±0.05

**Figure S1:** Relationship between mean leaf dry matter content (LDMC mean) and local species occurrence including *Sedum* *acre.* Lines represent model predictions of the averaged models for the standard GLMM (black line) and the one that incorporates the phylogenetic-relationships between species (red line, see Methods for details). Grey points refer to the data points. Please note that data points belong to different sampling sites and species. The influence of the mean of LDMC on species local occurrence appear unimodal (see Results Figure 1a).


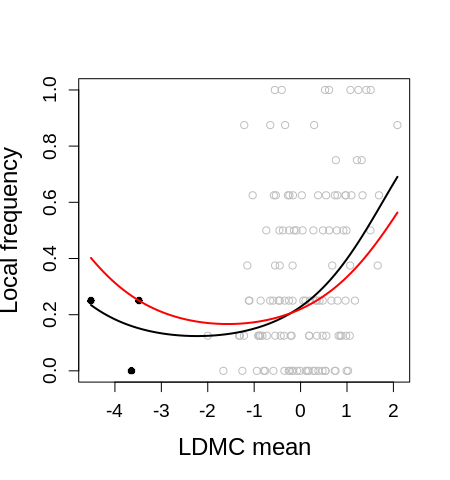


**Appendix S3**

**Table S3:** Species sampled and their local and regional occurrences. Local occurrence refers to the frequency of species across the eight subplots within the 10m x 10m (mean± standard deviation). Regional occurrence refers to the number of 10m x 10m plots that a species was found. The last column (# trait sampling) signifies at how many plots the species was randomly selected for trait measurements.

Species local occurrence regional occurrence # trait (mean±SD) sampling

*Achillea* *millefolium* agg. 3.83 ±3.31 16 6

*Agrimonia* *eupatoria* 1.25 ±0.96 10 4

*Allium* *vineale* 1.00 ±0.00 8 1

*Arrhenatherum* *elatius* 4.50 ±2.59 18 6

*Artemisia* *campestris* 1.00 ±0.82 13 7

*Brachypodium* *pinnatum* 6.50 ±2.12 3 2

*Bromus* *erectus* 7.67 ±0.58 4 3

*Carex* *hirta* 3.00 ±1.41 5 2

*Centaurea* *scabiosa* 1.50 ±0.71 14 2

*Centaurea* *stoebe* 1.00 ±1.41 10 2

*Dactylis* *glomerata* 0.50 ±0.71 15 2

*Daucus* *carota* 1.67 ±2.08 10 3

*Dianthus* *carthusianorum* 1.00 ±0.82 13 4

*Echium* *vulgare* 0.33 ±0.58 10 3

*Elymus* *repens* 2.00 ±0.00 9 1

*Falcaria* *vulgaris* 2.83 ±1.94 14 6

*Festuca* *brevipila* 5.75 ±2.63 14 4

*Festuca* *rubra* 4.00 ±0.00 15 1

*Filipendula* *vulgaris* 2.00 ±0.00 2 1

*Fragaria* *viridis* 3.50 ±2.08 7 4

*Galium* *album* 3.43 ±2.64 16 7

*Galium* *verum* 4.00 ±0.00 3 1

*Helichrysum* *arenarium* 0.00 ±0.00 8 2

*Helictotrichon* *pratense* 0.00 ±0.00 4 2

*Hieracium* *pilosella* 2.50 ±1.91 10 4

*Knautia* *arvensis* 2.00 ±0.00 9 2

*Medicago* *lupulina* 0.00 ±0.00 6 1

*Medicago* *sativa* agg. 2.40 ±1.82 7 5

*Origanum* *vulgare* 6.50 ±2.12 2 2

*Phleum* *phleoides* 3.43 ±1.72 12 7

*Picris* *hieracioides* 3.33 ±2.25 9 6

*Plantago* *lanceolata* 1.80 ±1.79 8 5

*Poa* *angustifolia* 0.00 ±0.00 20 1

*Potentilla* *incana* 3.50 ±3.54 7 2

*Potentilla* *reptans* 5.00 ±0.00 1 1

*Salvia* *pratensis* 1.00 ±0.00 5 1

*Sanguisorba* *minor* 2.67 ±2.52 7 3

*Scabiosa* *columbaria* 0.00 ±0.00 3 2

*Sedum* *acre* 1.33 ±1.15 10 3

*Senecio* *jacobaea* 0.00 ±0.00 13 1

*Silene* *nutans* 5.00 ±0.00 1 1

*Thymus* *pulegioides* 2.75 ±2.99 8 4

*Veronica* *chamaedrys* 1.00 ±0.00 8 1

**Appendix S4**

**Table S4**: Presentation of the best (lowest AICc) models that were used to get the averaged models (Tab. 1, main manuscript). The table shows the best models sorted by AIC_c_ for each analysis including the respective log-likelihood, AIC_c_ value and the parameter estimates. If no value is given for a specific parameter, the parameter was not included in the model. Please note that for the regional phylogenetic-corrected model, no intercept is estimated (Felsenstein 1985).

| **Intercept** | **Plant height** | | | **LDMC** | | | **SLA** | | | **df** | **logLik** | **AIC_c_** |
| --- | --- | --- | --- | --- | --- | --- | --- | --- | --- | --- | --- | --- |
|  | **CV** | **mean** | **mean²** | **CV** | **mean** | **mean²** | **CV** | **mean** | **mean²** |  |  |  |
|  |  |  |  |  |  |  |  |  |  |  |  |  |
| **Local model** | | | | | | | | | |  |  |  |
| -1.24 | 0.29 |  |  | 0.23 | 0.49 | 0.17 |  |  |  | 7 | -271.63 | 558.21 |
| -1.10 | 0.29 |  |  | 0.22 | 0.49 |  |  |  |  | 6 | -272.83 | 558.37 |
| -1.25 | 0.29 |  |  | 0.22 | 0.61 | 0.17 |  | 0.22 |  | 8 | -270.99 | 559.23 |
| -1.10 | 0.29 |  |  | 0.20 | 0.61 |  |  | 0.22 |  | 7 | -272.23 | 559.43 |
| -1.13 | 0.28 |  |  | 0.23 | 0.59 | 0.19 |  | 0.19 | -0.14 | 9 | -269.97 | 559.51 |
| -1.09 | 0.27 |  |  |  | 0.45 |  |  |  |  | 5 | -274.79 | 560.08 |
| -0.98 | 0.28 |  |  | 0.21 | 0.60 |  |  | 0.19 | -0.12 | 8 | -271.48 | 560.21 |
|  |  |  |  |  |  |  |  |  |  |  |  |  |
| **Local model, Phylo-corrected** | | | | | | | | | | | | |
| -1.23 | 0.36 |  |  | 0.26 | 0.36 | 0.20 |  |  |  | 7 | -281.59 | 578.13 |
| -1.11 | 0.36 |  |  | 0.25 | 0.36 |  |  |  |  | 6 | -283.24 | 579.19 |
|  |  |  |  |  |  |  |  |  |  |  |  |  |
|  |  |  |  |  |  |  |  |  |  |  |  |  |
| **Regional model** | | | | | | | | | | | | |
| 0.04 | -0.44 |  |  |  |  |  |  |  |  | 3 | -56.30 | 119.22 |
| 0.04 | -0.46 |  |  | 0.24 |  |  |  |  |  | 4 | -55.62 | 120.32 |
| 0.05 | -0.50 |  |  |  | -0.17 |  |  |  |  | 4 | -55.79 | 120.65 |
| 0.04 | -0.38 | 0.16 |  |  |  |  |  |  |  | 4 | -55.92 | 120.92 |
| -0.13 | -0.48 |  |  |  | -0.15 | 0.23 |  |  |  | 5 | -54.74 | 121.15 |
| 0.04 | -0.46 |  |  |  |  |  | 0.15 |  |  | 4 | -56.05 | 121.17 |
|  |  |  |  |  |  |  |  |  |  |  |  |  |
| **Regional model, Phylo-corrected** | | | | | | | | | | | | |
| - | -0.43 |  |  |  | -0.24 |  |  |  |  | 3 | -52.59 | 111.84 |
| - | -0.40 |  |  |  |  |  |  |  |  | 2 | -54.00 | 112.31 |
| - | -0.43 |  |  |  | -0.24 | -0.09 |  |  |  | 4 | -51.83 | 112.77 |
| - | -0.51 |  |  |  | -0.39 |  |  | -0.23 |  | 4 | -51.93 | 112.96 |
| - | -0.46 |  |  | -0.16 | -0.28 |  |  |  |  | 4 | -51.97 | 113.05 |
| - | -0.43 |  |  |  | -0.23 |  | -0.1 |  |  | 4 | -52.33 | 113.77 |
